# *In vitro* characterization of virulence factors among species of the *Candida parapsilosis* complex

**DOI:** 10.1590/0037-8682-0336-2019

**Published:** 2020-01-27

**Authors:** Fábio Silvestre Ataides, Carolina Rodrigues Costa, Andressa Santana Santos, Vivianny Aparecida Queiroz Freitas, Thaisa Cristina Silva, Ana Laura Sene Amâncio Zara, Rosália Santos Amorim Jesuino, Maria Rosário Rodrigues Silva

**Affiliations:** 1 Universidade Federal de Goiás, Instituto de Patologia Tropical e Saúde Pública, Goiânia, GO, Brasil.; 2 Universidade Federal de Goiás, Instituto de Ciências Biológicas II, Goiânia, GO, Brasil.

**Keywords:** Candida parapsilosis, Virulence factors, Biofilms, Cell adhesion, Hydrolytic enzymes

## Abstract

**INTRODUCTION:**

*Candida parapsilosis* complex species differ from each other with regard to their prevalence and virulence*.*

**METHODS:**

The hydrolytic enzyme activity, biofilm production, and adhesion to epithelial cells were analyzed in 87 *C. parapsilosis* complex strains.

**RESULTS:**

Among the studied isolates, 97.7%, 63.2%, and 82.8% exhibited very strong proteinase, esterase, and hemolysin activity, respectively. All the *C. parapsilosis* complex isolates produced biofilms and presented an average adherence of 96.0 yeasts/100 epithelial cells.

**CONCLUSIONS:**

Our results show that *Candida parapsilosis* complex isolates showed different levels of enzyme activity, biofilm production, and adhesion to epithelial cells.

Several species belonging to the genus *Candida* have emerged as important pathogens causing a broad variety of clinical infections. The *C. parapsilosis* complex (*C. parapsilosis sensu stricto*, *C. orthopsilosis*, and *C. metapsilosis*) are among the most common species of *Candida* responsible for nosocomial bloodstream infections and are an important cause of onychomycosis, mainly affecting the fingernails^1^.

Although members of the *C. parapsilosis* complex are closely related, their clinical prevalence and virulence are different. These virulence factors including the ability to secrete hydrolytic enzymes, adhesion to host epithelial cells, biofilm production capability, and hemolytic activity are considered important to the initiation and maintenance of their infections^1^ ^,^ ^2^. In this study, we evaluated the *in vitro* capacity of *C. parapsilosis sensu stricto*, *C. orthopsilosis,* and *C. metapsilosis* isolates to produce hydrolytic enzymes, to adhere to human buccal epithelial cells, and produce biofilm. 

In total, the 87 isolates identified were *C. parapsilosis sensu stricto* (n = 78), *C. orthopsilosis* (n = 5), and *C. metapsilosis* (n = 4), which included 54 candidemia cases and 33 onychomycosis cases obtained from the Laboratory of Mycology, Institute of Tropical Pathology and Public Health*,* Federal University of Goiás, from 2007 to 2012. These isolates were stored at -70°C in yeast extract peptone dextrose (YEPD) broth (Difco®) with 10% glycerol. Subcultivation was carried out on YEPD agar (Difco®) for 48 h at 37°C. The study was approved by the Bioethics Committee of the Clinical Hospital of the Federal University of Goiás (Protocol no. 065/2008). 

Aspartic proteinase (Sap) was assessed using a 10-µL aliquot of yeast suspension (10^7^ cells/mL), which was inoculated on the surface of bovine serum albumin (BSA) agar medium (pH 3.5) and incubated at 37°C for 7 days. For the phospholipase assay, 10 µL of yeast suspension (10^7^ cells/mL) was placed on the surface of agar medium containing egg yolk and incubated at 37°C for 4 days. To determine esterase activity, a 5-µL aliquot of a yeast suspension (10^7^ cells/mL) was placed on the surface of Tween opacity test medium and incubated at 37°C for 10 days^2^. 

Hemolytic enzyme activity was determined by inoculating 5 µL of yeast suspension (10^8^ cells/mL) on the surface of Sabouraud agar medium supplemented with 7% sheep blood and 3% glucose^2^. The plates were then incubated at 37°C for 48 h, and hemolysis was observed based on the presence of a translucent halo around the colony. A β-hemolytic *Staphylococcus aureus* strain (ATCC 6538) was used as the positive control. 

Measurements and calculations of hydrolytic enzyme activity (Pz) were performed as described by Price et al.^3^ **.** Pz coefficients of the analyzed *Candida* strains were grouped into five classes: very strong (Pz < 0.69), strong (Pz = 0.70-0.79), mild (Pz = 0.80-0.89), weak (Pz = 0.90-0.99), and negative (Pz = 1.0). 

For biofilm production, *C. parapsilosis* complex isolates at a final concentration of 1.0 × 10^6^ cells/mL, were suspended in RPMI 1640 broth supplemented with L-glutamine and buffered with MOPS. Aliquots of 100 µL were then inoculated into flat-bottom 96-well microtiter plates and incubated for 48 h at 37°C. After biofilm formation, the wells were washed thrice with PBS to remove non-adhered cells. Semiquantitative measurements of biofilm production were obtained via the XTT reduction assay^4^. The absorbance of XTT assays was read spectrophotometrically (Tp-Reader-Basic, Thermo Plate) at 492 nm. Biofilm production was measured based on optical density (OD) > 0.2**.**

For adhesion assay, 500 µL of each yeast suspension (10^7^ yeast/mL) were mixed with 500 μL of human buccal epithelial cell (HBEC) suspension (2 × 10^5^ cells/mL) and incubated at 37°C with agitation for 1 h. The mixture was subsequently filtered through a 20 μm-pore-size membrane filter and transferred to a slide by pressing the filter paper against it. After fixing the cells with methanol, they were subjected to Gram staining, and the number of cells that adhered to 100 HBECs was counted^5^ **.**

All assays were performed in triplicate for each isolate on different days, and the mean values were determined. Statistical analysis employed Mann-Whitney and Kruskal-Wallis tests when appropriate (non-parametric tests). The significance level was set at 0.05. The data were analyzed using IBM SPSS Statistics version 20.

Most of the 87 isolates of *C. parapsilosis* presented very strong enzymatic activity (Pz > 0.69) for proteinase (n = 85; 97.7%), esterase (n = 55; 63.2%), and hemolysin (n = 72; 82.8%). However, phospholipase activity was negative in 89.8% (n = 78) of strains (Table 1). 

**TABLE 1:** Enzymatic profiles of *C. parapsilosis* species complex isolates (n = 87) from blood and nail samples. Goiania-GO, Brazil.

Enzymatic activity (Pz*)	*C. parapsilosis*		*C. orthopsilosis*	*C. metapsilosis*		Total
	*sensu stricto*		**blood**	**blood**	**nails**	**n (%)**
	blood	nails	(n = 5)	**(n = 2)**	**(n = 2)**	(n = 87)
	(n = 47)	(n = 31)				
Proteinase						
Very strong	47	31	5	2	-	85 (97.7)
Negative	-	-	-	-	2	2 (2.3)
Phospholipase						
Very strong	-	5	-	2	-	7 (8.0)
Strong	-	1	-	-	-	1 (1.1)
Mild	-	1	-	-	-	1 (1.1)
Negative	47	24	5	-	2	78 (89.8)
Esterase						
Very strong	29	19	4	2	1	55 (63.2)
Strong	13	6	-	-	-	19 (21.8)
Negative	5	6	1	-	1	13 (15.0)
Hemolysin						
Very strong	42	23	4	2	1	72 (82.8)
Strong	3	4	-	-	1	8 (9.2)
Mild	2	4	-	-	-	6 (6.9)
Weak	-	-	1	-	-	1 (1.1)

*Pz = Value obtained by dividing the diameter of the colony (mm) by the total diameter of the colony including the precipitation zone (mm). Pz ≤ 0.69: very strong; Pz = 0.70-0.79: strong; Pz = 0.80-0.89: mild; Pz = 0.90-0.99: weak; Pz = 1: negative.

C. *parapsilosis sensu stricto* isolates produced mean Pz of esterase activity higher than the others (p = 0.015), especially in blood samples (p = 0.025) (Table 2). 

**TABLE 2:** Values of enzymatic activity (Pz mean) by *C. parapsilosis* species complex isolates, according to enzyme and anatomical site (blood and nails). Goiânia-GO, Brazil.

Enzymes	***Candida parapsilosis* complex**			p-value***
	*C. parapsilosis sensu stricto*	*C. orthopsilosis*	*C. metapsilosis*	
	Pz* mean ± SD**	Pz mean ± SD	Pz mean ± SD	
Total (n = 87)	n = 78	n = 5	n = 4	
Proteinase	0.304 ± 0.084	0.316 ± 0.096	0.385 ± 0.078	0.251
Phospholipase	0.664 ± 0.075	-	0.630 ± 0.042	0.551
Esterase	0.559 ± 0.130	0.466 ± 0.303	0.383 ± 0.076	0.015****
Hemolysin	0.592 ± 0.100	0.572 ± 0.147	0.552 ± 0.147	0.599
Blood (n = 54)	n = 47	n = 5	n = 2	
Proteinase	0.328 ± 0.096	0.316 ± 0.096	0.385 ± 0.078	0.590
Phospholipase	-	-	0.630 ± 0.042	-
Esterase	0.562 ± 0.132	0.466 ± 0.303	0.350 ± 0.070	0.025****
Hemolysin	0.580 ± 0.087	0.572 ± 0.147	0.450 ± 0.070	0.122
Nails (n = 33)	n = 31	n = 0	n = 2	
Proteinase	0.263 ± 0.039	-	-	-
Phospholipase	0.664 ± 0.075	-	-	-
Esterase	0.554 ± 0.128	-	0.450 ± 0	0.345
Hemolysin	0.609 ± 0.117	-	0.655 ± 0.134	0.650

*Pz = Value obtained by dividing the diameter of the colony (mm) by the total diameter of the colony including the precipitation zone (mm). **SD: Standard deviation. ***Kruskal-Wallis test. ****Bold p-values indicate statistically significant differences (p < 0.05).

Proteinase activity was higher in isolates from blood than in isolates from nails (p < 0.001) (Figure 1A).

All *C. parapsilosis* complex isolates could produce biofilms. Although the biofilms formed by *C. metapsilosis* isolates from blood had a higher mean OD than nail isolates, no statistically significant difference was detected (p = 1.000) (Figure 1B). 

The *C. parapsilosis* complex isolates exhibited high adherence to HBEC, with no statistically significant differences between isolates or anatomical sites (Figure 1C). The greatest number of adhered yeasts was observed in case of *C. parapsilosis sensu stricto* (100.2 ± 88.7), followed by *C. metapsilosis* (90.8 ± 28.9), and *C. orthopsilosis* (38.0 ± 19.3). 

**FIGURE 1:**
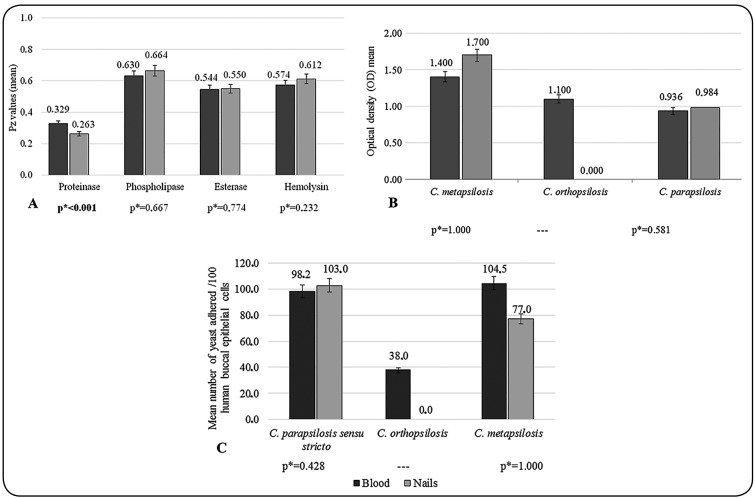
Differences in the mean enzymatic activities of phospholipase, proteinase, esterase, and hemolytic factors, the metabolic activity of biofilms, and the mean number of yeasts that adhered to 100 HBECs between different sources and species of the *C. parapsilosis* complex. **(A)** Values of enzymatic activity (Pz mean), according to enzyme and anatomical site (blood and nails). **(B)** Optical density (OD) mean of the biofilms formed, according to anatomical site (blood and nails). **(C)** Mean number of yeast cells adhered/100 human buccal epithelial cells, according to anatomical site (blood and nails). *Mann-Whitney test (p < 0.05).

Very strong *in vitro* proteinase activity was detected in all isolates of *C. parapsilosis sensu stricto* and *C. orthopsilosis*, whereas only two isolates of *C. metapsilosis* showed positive enzymatic activity. Treviño-Rangel et al.^2^ also observed proteinase activity in three species of the complex. Several studies have shown that *Candida parapsilosis* complex species express different proteinase activities. Silva et al.^6^ found positive protease activity in 37.7% isolates of *C. parapsilosis sensu stricto*; however, only 7.8% of the isolates revealed high enzymatic activity. Furthermore, none of the *C. metapsilosis* and *C. orthopsilosis* isolates exhibited protease activity in that study. 

Some studies on the virulence of *Candida* species verified that few isolates of the *C. parapsilosis* complex have proteinase activity, regardless of whether the isolation site was nail or blood^1^ ^,^ ^7^. Our research identified significantly higher enzymatic activity of proteinase in isolates from blood than from nails (Figure 1A). This difference in proteinase activity between nail and blood isolates suggests that this enzyme may be associated with increased adhesion and invasion capacity in the bloodstream. 

Few isolates in our study produced phospholipase (10.3%), which was also found by Dagdeviren et al.^5^ and Silva et al.^6^, who verified phospholipase activity in 15.1% and 8.7% of *C. parapsilosis* isolates, respectively. However, some isolates of the *C. parapsilosis* complex may show high production of phospholipase. Among these *C. orthopsilosis* has the highest phospholipase activity, with up to 69% of isolates exhibiting very high enzymatic activity^2^. 

In contrast to the observations for phospholipase, a high percentage of our isolates could produce esterase. High esterase activity was found by Pakshir et al.^8^ in 56.5% of the *C. parapsilosis* isolates studied, but Akyol & Cerikçioğlu^9^ detected this activity in only 1.88% of the examined isolates. In the present study, esterase activity was similar among members of the *C. parapsilosis* complex with 85.8% of *C. parapsilosis sensu stricto*, 80% of *C. orthopsilosis,* and 75% of *C. metapsilosis* isolates. Different results were obtained by Treviño-Rangel et al.^2^, who observed esterase production in 13.3% of *C. parapsilosis* and 66.6% of *C. orthopsilosis* isolates studied but not in any *C. metapsilosis* isolates.

We identified hemolytic activity in 100% of the *C. parapsilosis* complex isolates (**Table 1**). Different results were found by Abi-Chacra et al.^10^, who detected weak hemolytic activity in all the *C. parapsilosis* complex isolates examined. Similarly, Treviño-Rangel et al.^2^ detected hemolysis ability among the isolates studied, but did not report high activity (Pz < 0.69), as observed in our isolates. 

As noted above, these differences in the enzymatic activity of phospholipase and esterase, and hemolytic capacity among of the *C. parapsilosis* complex isolates *in vitro* can be explained by the biological variation between isolates according to the infected anatomic site. Nail isolates had higher Pz mean values ​​than blood isolates, although this difference was not significant. This difference can be due to the evolution of the infection, as most cases of onychomycosis were characterized by chronic evolution, whereby more physiological conditions of the etiological agent were required to maintain its pathogenic capacity. Other hydrolytic enzymes with unknown activities against different relevant substrates specific to human cutaneous infections such as onychomycosis, unlike those used in the present study, could further our understanding of this biological variation in enzymatic secretion between isolates according to the isolation site.

In the present work, *C. parapsilosis* complex isolates presented a high affinity for human buccal epithelial cells *in vitro*, with adherence of 96.0 yeasts per 100 epithelial cells. Costa et al.^11^ verified increased adherence capacity, with an average value of 155.3 yeasts whereas Dagdeviren et al.^5^ and Lima-Neto et al.^12^ reported lower numbers of adhered yeasts, at 54.9 and 25.6 on average, respectively. Thus, intraspecies variation in adherence to human buccal epithelial cells is evident among *C. parapsilosis* isolates, with values up to more than 150 fungal cells per 100 epithelial cells. 

Although Németh et al.^13^ demonstrated that *C. metapsilosis* is the least virulent species of the *psilosis* group, our results showed no statistically significant differences in HBEC adherence ability within the *C. parapsilosis* complex isolates. However, *C. metapsilosis* isolated from blood had greater ability to adhere than the other strains, which may be because *C. parapsilosis* interacts with different mammalian nails.

This study confirmed that all the examined *C. parapsilosis* complex isolates could produce a biofilm. Similarly, Lattif et al.^14^ reported that all clinical isolates of the *C. parapsilosis* complex obtained from silicone disks of catheters could form biofilms. Some authors^14^ ^,^ ^15^reported contrasting results regarding the ability of these three species of the complex to form biofilms. Song et al.^15^ found that *C. orthopsilosis* and *C. metapsilosis* did not form biofilms, whereas Lattif et al.^14^ verified that all three species were capable of biofilm production. In terms of our findings, even though *C. metapsilosis* are considered the less virulent, these isolates had higher adhesion capacity and biofilm formation than *C. parapsilosis stricto sensu* and *C. orthopsilosis*.

In the present work, comparison of biofilm-forming abilities among isolates did not reveal any significant differences between the three species of the *C. parapsilosis* complex. However, according to the anatomical site of isolation, *C. parapsilosis stricto sensu* isolated from nails showed higher mean values of adhered yeasts and O.D. in biofilm formation than blood isolates. 

In conclusion, the proteinase activity of *C. parapsilosis* isolates was higher, but a statistical evaluation could not be performed due to the small number of species obtained. These results suggest that the high adherence ability of species participates in initial biofilm formation. The association of adherence ability to buccal cells with biofilm formation can be an important parameter to differentiate invasive from non-invasive strains. Screening phospholipase production in biofilm-forming isolates can also an important parameter to distinguish invasive strains from noninvasive colonizers. The data presented in this report reinforce the necessity of assessing the phenotypic differences in the *C. parapsilosis* complex, including those related to the expression of virulence attributes that play relevant roles in establishing the infectious process.
